# Risk of sarcopenia in community dwelling older adults in Kuwait

**DOI:** 10.3389/fpubh.2025.1703711

**Published:** 2026-01-16

**Authors:** Latifah Alenezi, Maath Alhaddad, Ali N. Ali

**Affiliations:** 1 Physical Therapy Department, Faculty of Allied Health Sciences, Kuwait University, Kuwait City, Kuwait; 2 School of Medicine and Population Health, University of Sheffield, Sheffield, United Kingdom

**Keywords:** Kuwait, older adults, risk, SARC-F, sarcopenia

## Abstract

**Introduction:**

Sarcopenia is characterized by a loss of skeletal muscle mass, strength, and/or physical performance, and is associated with numerous adverse health outcomes. Data on the risk of sarcopenia in Arabic speaking countries is lacking, in particular in Kuwait, and this study aimed to provide initial estimates of sarcopenia risk. Given that sarcopenia predicts frailty, we also examined how population characteristics interact with sarcopenia risk and key frailty determinants, including mobility, strength, independence, and falls risk.

**Methods:**

This cross-sectional study included community-dwelling older adults in Kuwait City. In a single session, data collected included socio-demographics; sarcopenia risk using the SARC-F screening tool; independence in daily living using the Physical Self-Maintenance Scale (PSMS); fear of falling using the Short Falls Efficacy Scale-International (Short FES-I); frailty using the Fatigue, Resistance, Ambulation, Illnesses, and Loss of weight (FRAIL) scale; strength using handgrip strength and the Five Times Sit-to-Stand Test (FTSTS); and mobility using the 4-meter gait speed test and Timed Up and Go (TUG) test. Participants were classified as at risk (SARC-*F* ≥ 4) or not at risk (< 4). Associations were analyzed using Chi-square and ANOVA. Logistic regression identified factors linked to sarcopenia and frailty, treating the persons without frailty as the reference group. Model fit was assessed by McFadden’s R^2^.

**Results:**

A total of 92 older adults participated in the study. Of these, 29 (31.5%) were at risk of sarcopenia (SARC-*F* ≥ 4). Females were significantly more likely to exhibit sarcopenia risk (47.4%) than males (20.4%) (*p* = 0.006). Sarcopenia risk was significantly associated with lower independence, slower gait speed, and higher age, FRAIL scores, TUG, and 5xSTS values. Logistic regression showed that female gender and advanced age (70–79 years) were significant predictors of sarcopenia risk. Frailty was present in 18.5% of participants and was strongly associated with multimorbidity (>3 chronic conditions). Concern about falling was common, with 73.9% reporting moderate to high concern.

**Conclusion:**

The risk of sarcopenia among older adults in Kuwait may be high. Accurate risk estimates are needed, involving muscle mass assessments and exploration of risk factors in order to implement effective screening and intervention services.

## 1 Introduction

According to the Asian Working Group for Sarcopenia (AWGS 2019), sarcopenia is defined by low muscle mass, low muscle strength, and/or poor physical performance (1), is an age-related progressive condition gaining attention over the last 30 years due to its strong links with frailty and poor health outcomes (2). Older adults with sarcopenia - are at increased risk of falls and fractures (3), hospitalisation (4), depression (5), functional decline (6), and all-cause mortality (7) compared to older adults without sarcopenia. The global prevalence of sarcopenia ranges from 10 to 27% (8) and is increasing due to aging populations in both developed and developing countries. The societal and economic impact of sarcopenia is significant; in the US, sarcopenia accounted for approximately 1.5% of total health spending ($18.5 billion) in 2004 (9), and this is likely higher today.

Recognition of sarcopenia’s impact occurred alongside the development of comprehensive tools for screening and diagnosis, which aim to detect the condition and identify individuals at risk (10). Initially developed in European populations, tools such as the SARC-F questionnaire, physical performance tests, and functional scales have been used and validated in many countries worldwide. Understanding sarcopenia’s risk is a critical step in developing screening services, which could have substantial benefits as early interventions, such as resistance training programs (11) and nutritional optimization (12), may delay or even reverse sarcopenia in affected individuals or those at risk.

International consensus statements (e.g., AWGS 2019; EWGSOP2) emphasize the clinical and public-health significance of early detection and targeted interventions. Despite this, there is a marked lack of data from Arabic-speaking countries, and, to our knowledge, no estimates of sarcopenia risk among community-dwelling older adults in Kuwait.

Research problem: The burden and correlates of sarcopenia risk in Kuwait are unknown, as are the patterns of association between sarcopenia risk and functional determinants (mobility, balance, strength, independence) and psychosocial factors (fear of falling). Kuwait’s demographic and sociocultural context—including high obesity prevalence, sedentary occupations, lower habitual physical activity, and limited sun exposure among women—may possibly elevate sarcopenia risk and modify related functional outcomes (13). Without country-specific evidence, health systems lack a foundation for implementing proactive screening pathways and rehabilitation strategies tailored to local needs.

Therefore, this study aimed to provide initial estimates of sarcopenia risk in a sample of community-dwelling older adults in Kuwait and to examine how population characteristics and key frailty determinants (mobility, strength, independence, and falls risk) interact with sarcopenia risk.

### 1.1 Study hypothesis

Grounded in international evidence and prior literature (1, 3, 6, 7, 10), we hypothesized that: (1) older adults in Kuwait have a higher risk of developing sarcopenia compared to globle average; (2) Females aged 70 years or above are at higher risk of developing sarcopenia compared to male counterparts; (3) sarcopenia risk would be associated with poorer functional performance (slower gait speed, prolonged Timed Up and Go [TUG], longer Five-Times Sit-to-Stand [5xSTS]) and lower independence in activities of daily living; and (4) sarcopenia risk would align with higher FRAIL scores and greater concern about falling.

## 2 Methods

### 2.1 Design

This was a multi-centre, cross-sectional observational study with recruitment.

### 2.2 Population and setting

Participants were community-dwelling older adults aged 65 and above, recruited from 5 different elder community groups and the general public in Kuwait City. Inclusion criteria were: age ≥65 years and ability to ambulate independently within the household, with or without assistive devices. Exclusion criteria included acute illness, medical conditions associated with cachexia (e.g., cancer, heart failure), severe cognitive impairment, and inability to read or understand Arabic. Screening was based on self-reported medical history and informal clinical indicators assessed by trained physiotherapists. Questionnaires were translated into Arabic by bilingual experts, and verbal assistance was provided by physiotherapists to ensure accessibility.

### 2.3 Data collection tools

#### 2.3.1 SARC-F

SARC-F is a reliable and valid screening tool for sarcopenia (14), comprising five components: strength, assistance walking, rising from a chair, climbing stairs, and falls. Scores range from 0 to 10, with a higher score indicating a greater risk of sarcopenia. Participants were categorized as SARC-F positive (score ≥ 4) or negative (score < 4). While the SARC-F has not been formally validated in an Arabic translation, such translations have been used in prior studies (15).

#### 2.3.2 Physical Self-Maintenance Scale

The PSMS is a reliable and valid instrument for assessing independence in activities of daily living (ADLs) in older adults. It comprises two sections: the first includes six items related to basic ADLs, and the second includes eight items related to instrumental ADLs (IADLs). Each item is rated on a five-point scale, where 0 indicates complete dependence and 5 indicates complete independence. In this study, only the first section was used to assess independence in six activities of daily living (ADL) for older adult populations (16).

#### 2.3.3 Short version of the Falls Efficacy Scale – International

The Short FES-I is a validated tool used to measure fear of falling (17), composed of 7 questions that assess concern for falls during differing ADLs, scoring each item 1 (no concern) to 4 (very concerned). Scores were trichotamised: 7–8 = low concern; 9–13 = moderate concern; 14–28 = great concern. The Arabic version was utilized in this study (18).

#### 2.3.4 The Fatigue, Resistance, Ambulation, Illnesses, and Loss of weight scale (FRAIL)

A short assessment tool assessing frailty (19), containing 5 questions directed at components of the Cardiovascular Health Study Frailty Index (20), relating to fatigue, resistance, ambulation, illnesses and weight loss. Presence of these issues scores a single point. Individuals are considered robust (score 0), having pre frailty (score 1–2) or having frailty (score 3–5). The Arabic version, which has demonstrated validity and reliability, was used in this study (21).

#### 2.3.5 4-meter gait speed

The 4-meter gait speed test is a validated measure of mobility and lower limb strength. A 4-meter path was marked with an additional 1 meter at each end to allow for acceleration and deceleration. Participants were instructed to walk at their usual pace while the time to cover the central 4 meters was recorded using a stopwatch. Assistive devices were permitted, but no physical assistance was allowed. A gait speed of ≤0.8 m/s was considered indicative of slow walking speed (22).

#### 2.3.6 Timed Up and Go test (TUG)

The TUG test is a reliable and valid tool for assessing mobility and fall risk in older adults. Participants wore their usual footwear and used assistive devices if needed. From a seated position in a chair with armrests, they were instructed to stand up, walk 3 meters, turn around, return, and sit down (23). The time taken to complete the task was recorded. Those taking > 20s are considered fulfilling criteria for reduced physical performance and >13.5 s increased risk of falls (10, 24).

#### 2.3.7 Five-Times Sit-to-Stand test (5xSTS)

The 5xSTS test evaluates lower extremity strength, balance, and fall risk. Participants sat in a standard chair with arms crossed over the chest. Upon the command “go,” they stood up and sat down five times as quickly as possible. The time taken to complete the task was recorded. Normative values for this test are 11.4 s for individuals aged 60–69 years, 12.6 s for those aged 70–79 years, and 14.8 s for those aged 80–89 years. Longer times were considered indicative of increased fall risk (25).

#### 2.3.8 Hand grip strength

Grip strength was measured using an electronic hand dynamometer (TAKAI Scientific, Japan) according to standardized procedures (26); < 27Kg and < 16Kg indicated criteria for reduced muscle strength for males and females, respectively (10).

#### 2.3.9 Defining risk of sarcopenia

A diagnosis of sarcopenia could not be confirmed in this study because muscle mass was not quantitatively assessed using methods such as BIA, DEXA, or MRI. For the purposes of this exploratory analysis, risk of sarcopenia was defined as SARC-*F* ≥ 4. Among those at risk, probable sarcopenia was defined as participants scoring ≥ 4 on the SARC-F questionnaire combined with either reduced handgrip strength or prolonged Five-Times Sit-to-Stand performance. Participants who additionally demonstrated prolonged TUG and 4-Meter Gait Speed were classified as having probable severe sarcopenia.

#### 2.3.10 Rationale for assessment instruments

The selection of assessment instruments was guided by the study’s objective to estimate sarcopenia risk and examine its functional and psychosocial consequences among older adults in Kuwait. In line with the expanded study aim to explore the interaction of population characteristics on sarcopenia risk and related determinants of frailty, these measures were included to provide a comprehensive understanding of vulnerability, mobility, and functional decline in this population. The Frailty Scale was included to evaluate frailty status, which is clinically relevant because frailty and sarcopenia share overlapping characteristics such as fatigue, reduced physical activity, and muscle weakness. Assessing frailty provides a broader understanding of vulnerability and functional decline in this population. The Physical Self-Maintenance Scale was used to measure independence in activities of daily living, an essential functional domain often compromised by sarcopenia-related reductions in muscle strength and physical performance. The Falls Efficacy Scale – International was incorporated to assess fear of falling, a psychological factor that influences mobility, activity participation, and quality of life in older adults with impaired balance and strength.

Physical performance tests were selected based on international consensus guidelines for sarcopenia diagnosis. The Four-Meter Gait Speed Test is a validated measure of mobility and lower limb strength, while the Timed Up and Go Test evaluates dynamic balance, functional mobility, and fall risk. The Handgrip Strength Test is widely recognized as a core indicator of overall muscle strength and is integral to sarcopenia diagnostic criteria. Finally, the Five-Times Sit-to-Stand Test was included to assess lower limb strength and functional capacity. Although normative values for handgrip strength and sit-to-stand performance are not specific to the Arabic population, these tests remain clinically relevant and their limitations are acknowledged in this study.

### 2.4 Procedure

Ethical approval was obtained from the Institutional Review Boards of Kuwait University- Health Science Centre (Ref. 126/2022). All procedures were conducted in accordance with ethical standards and approved by the relevant institutional review board. Participants were recruited using a convenience sampling method from five elder community groups and public venues across Kuwait City. Recruitment was facilitated through outreach efforts, including flyers, word-of-mouth, and social media. The study employed a convenience sampling approach due to the exploratory nature of the study and logistical constraints commonly encountered in community-based research. Participants signed an in-formed consent form to ensure their understanding of the purpose of this research study as well as the confidentiality of their responses. Upon obtaining informed consent, participants underwent a series of assessments conducted by trained physiotherapists. All assessments were completed in a single session following a structured protocol. Participants first completed a demographic questionnaire (age, sex, marital status, education, smoking status), followed by self-reported scales, including SARC-F, Physical Self-Maintenance Scale, the Short Falls Efficacy Scale-International, the Fatigue, Resistance, Ambulation, Illnesses, and Loss of weight scale. Subsequently, physical performance tests were administered in the following order: Handgrip Strength, Five-Times Sit-to-Stand, Timed Up and Go, and 4-Meter Gait Speed. The entire assessment session lasted approximately 45–60 min per participant, depending on individual mobility and comprehension. All assessments were conducted by trained physiotherapists fluent in Arabic to ensure consistency and participant understanding. All instruments were administered in Arabic, with English versions used only when participants did not understand Arabic (such as non-Kuwaiti participants whose first language was not Arabic). Data collection began on May, 2022 and ended on June, 2023.

### 2.5 Data analysis

The characteristics of the participants were summarized using descriptive statistics. Frequencies and percentages were employed for categorical variables, while means and standard deviations were employed for continuous variables. Participants were categorized into 2 groups according to sarcopenia risk (SARC-*F* ≥ 4 = at risk; SARC-*F* < 4 = not at risk). Categorical variables between groups were compared using the Chi-square test among groups with varying levels of the group. ANOVA was implemented for continuous variables. Binomial logistic regression to identify factors associated with the risk of sarcopenia. The model’s fit was evaluated using McFadden’s R^2^. The results were statistically significant at a *p*-value level of less than 0.05. All analyses were conducted using the JAMOVI software version 2.3.28.

## 3 Results

Table 1 shows the demographic characteristics of the participants. The study was based on a sample of 92 participants, among which 54 were males and 38 were females. More than half of the participants were from the age-group 70–79 years (54.3%), while 40.2% were from the age-group 60–69, and only 5.4% were from the age-group 80–89. The majority of the participants were married (81.5%) and lived in a nuclear family (88.0%).

**Table 1:** Demographic characteristics.

Variables	*N =* 92	%
Gender		
Female	38	41.3%
Male	54	58.7%
Age group		
60–69	37	40.2%
70–79	50	54.3%
80–89	5	5.4%
Marital status		
Married	75	81.5%
Not married	2	2.2%
Divorced	7	7.6%
Widowed	8	8.7%
Education		
Below high school	47	51.1%
High school	9	9.8%
Diploma	13	14.1%
Bachelor	14	15.2%
Post graduate certificate	9	9.8%
Living with		
Nuclear family	81	88.0%
Lives alone	2	2.2%
Others	2	2.2%
Extended family	7	7.6%
Health status		
Excellent	10	10.9%
Very good	25	27.2%
Good	31	33.7%
Fair	25	27.2%
Poor	1	1.1%
Smoker		
Yes	11	12.0%
No	81	88.0%
Dominant hand		
RT	85	92.4%
LT	7	7.6%
Chronic disease		
No	7	7.6%
One	22	23.9%
Two	26	28.3%
More than 3	37	40.2%
BMI		
Mean (SD)	30.3 (5.2)	–
Range	21.0–47.0	–

RT, Right hand; LT, Left hand; BMI, Body Mass Index; SD, Standard Deviation.

Participants were assigned to groups based on SARC-F scores (SARC-*F* ≥ 4 = 0, SARC-F < 4 = 1). From the whole sample (*n =* 92), 29 participants identified as at risk of sarcopenia (SARC-F ≥ 4), whereas 63 participants were not at risk (SARC-*F* < 4). Participants were also classified as persons without frailty (score = 0), persons with pre-frailty (score = 1–2), or persons with frailty (score = 3–5) based on the total FRAIL scale score.

Regarding education, 51.1% left formal education before high school, 15.2% completed a bachelor degree and 14.1% diploma. Among the total participants, 10.9% reported the health status as excellent, 27.2% as very good, 33.7% as good, and 27.2% as fair. Prevalence of smoking was low at 12 and 92.4% were right-handed. Chronic diseases were prevalent, with 40.2% reporting more than three conditions. The mean BMI (+/− SD) of the respondents was 30.3 ± 5.2.

Table 2 shows the distribution of sarcopenia risk, frailty status, functional performance, and fall concern among the study participants. Among the total participants, around one-third of the participants were classified as being at risk of sarcopenia (SARC-F positive), while 68.5% were classified as not at risk. Regarding Frailty category, 18.5% were persons with frailty, 48.9% were persons with pre-frailty and 32.6% were persons without frailty. Grip strength was reduced in 53.3%, while 5xSTS demonstrated abnormal readings (weakness of the legs and increased falls risk) in 82.6%. In the TUG, 48.9% demonstrated abnormally increased times, and 41.3% demonstrated slow walking speeds in the 4MGS test. Fear of falling was prevalent, with 43.5% of participants reporting great concern, and 30.4% moderate concern, with only 26.1% reporting low concern. The PSMS revealed that 42.4% of the participants were dependent.

**Table 2:** Prevalence of sarcopenia risk, frailty, functional performance, and fall concern among older adults in Kuwait (*N =* 92).

Variables	Overall (*N =* 92)	%
Sarcopenia		
Negative	63	68.5%
Positive	29	31.5%
Frailty		
Persons without frailty	30	32.6%
Persons with pre frailty	45	48.9%
Persons with frailty	17	18.5%
Handgrip strength (kg)		
Within normal	43	46.7%
Weak	49	53.3%
Timed Up and Go (s)		
No risk of fall	47	51.1%
Risk of fall	45	48.9%
Gait speed (4-Meter Walk Test, m/s)		
Slow walking speed	38	41.3%
Good walking speed	54	58.7%
Five Time Sit-to-Stand (5xSTS, s)		
No risk of fall	16	17.4%
Risk of fall	76	82.6%
Physical Self-Maintenance Scale		
Dependent	39	42.4%
Independent	53	57.6%
Short Falls Efficacy Scale		
Low concern	24	26.1%
Moderate concern	28	30.4%
Great concern	40	43.5%

Table 3 shows the results of *χ*^2^ test that was conducted to examine associations between sociodemographic, health, and functional variables with sarcopenia risk. Sarcopenia risk was associated with female gender, age category, lower education status, reported health status, and chronic disease burden (> 3 health conditions).

**Table 3:** Associations between sociodemographic, health, and functional variables and sarcopenia risk among older adults in Kuwait (*N =* 92).

Variables	Negative (*N =* 63)	Positive (*N =* 29)	Total (*N =* 92)	*p*-value
Gender				0.006^1^
Female	20.0 (31.7%)	18.0 (62.1%)	38.0 (41.3%)	
Male	43.0 (68.3%)	11.0 (37.9%)	54.0 (58.7%)	
Age group				0.007^1^
60–69	32.0 (50.8%)	5.0 (17.2%)	37.0 (40.2%)	
70–79	29.0 (46.0%)	21.0 (72.4%)	50.0 (54.3%)	
80–89	2.0 (3.2%)	3.0 (10.3%)	5.0 (5.4%)	
BMI				0.845^2^
Mean (SD)	30.2 (4.6)	30.4 (6.2)	30.3 (5.2)	
Range	22.0–43.0	21.0–47.0	21.0–47.0	
Marital status				0.357^1^
Married	52.0 (82.5%)	23.0 (79.3%)	75.0 (81.5%)	
Not married	2.0 (3.2%)	0.0 (0.0%)	2.0 (2.2%)	
Divorced	3.0 (4.8%)	4.0 (13.8%)	7.0 (7.6%)	
Widowed	6.0 (9.5%)	2.0 (6.9%)	8.0 (8.7%)	
Education				0.050^1^
Below high school	30.0 (47.6%)	17.0 (58.6%)	47.0 (51.1%)	
Diploma	11.0 (17.5%)	2.0 (6.9%)	13.0 (14.1%)	
Bachelor	12.0 (19.0%)	2.0 (6.9%)	14.0 (15.2%)	
High school	3.0 (4.8%)	6.0 (20.7%)	9.0 (9.8%)	
Post graduate certificate	7.0 (11.1%)	2.0 (6.9%)	9.0 (9.8%)	
Living with				0.150^1^
Nuclear family	56.0 (88.9%)	25.0 (86.2%)	81.0 (88.0%)	
Lives alone	2.0 (3.2%)	0.0 (0.0%)	2.0 (2.2%)	
Others	0.0 (0.0%)	2.0 (6.9%)	2.0 (2.2%)	
Extended family	5.0 (7.9%)	2.0 (6.9%)	7.0 (7.6%)	
Health status				< 0.001^1^
Excellent	10.0 (15.9%)	0.0 (0.0%)	10.0 (10.9%)	
Very good	21.0 (33.3%)	4.0 (13.8%)	25.0 (27.2%)	
Good	23.0 (36.5%)	8.0 (27.6%)	31.0 (33.7%)	
Fair	9.0 (14.3%)	16.0 (55.2%)	25.0 (27.2%)	
Poor	0.0 (0.0%)	1.0 (3.4%)	1.0 (1.1%)	
Smoker				0.289^1^
Yes	6.0 (9.5%)	5.0 (17.2%)	11.0 (12.0%)	
No	57.0 (90.5%)	24.0 (82.8%)	81.0 (88.0%)	
Dominant hand				0.502^1^
RT	59.0 (93.7%)	26.0 (89.7%)	85.0 (92.4%)	
LT	4.0 (6.3%)	3.0 (10.3%)	7.0 (7.6%)	
Chronic disease				0.009^1^
No	6.0 (9.5%)	1.0 (3.4%)	7.0 (7.6%)	
One	17.0 (27.0%)	5.0 (17.2%)	22.0 (23.9%)	
Two	22.0 (34.9%)	4.0 (13.8%)	26.0 (28.3%)	
More than 3	18.0 (28.6%)	19.0 (65.5%)	37.0 (40.2%)	

BMI: Body Mass Index. RT: Right hand; LT: Left hand. ^1^Pearson’s Chi-squared test. ^2^Linear Model ANOVA.

Table 4 shows how sociodemographic, physiological, and functional performance indicators are spread out across different levels of frailty. There was a strong link between gender and frailty (*p* < 0.001). Most of the participants with frailty were women (82.4%), but most of the persons without frailty (73.3%) and persons with pre-frailty (64.4%) participants were men. Age was strongly linked to frailty status (*p* = 0.016). Most adults between the ages of 70 and 79 were persons with pre-frailty (66.7%), while most participants between the ages of 60 and 69 were persons without frailty (63.3%). Additionally, marital status (*p* = 0.045) and education (*p* = 0.043) also found to be significantly associated with frail categories.

**Table 4:** Distribution of sociodemographic, health, and functional characteristics by frailty status among older adults in Kuwait (*N =* 92).

Variables	Persons without frailty (*N =* 30)	Persons with pre frailty (*N =* 45)	Persons with frailty (*N =* 17)	Total (*N =* 92)	*p*-value
Gender					< 0.001^1^
Female	8.0 (26.7%)	16.0 (35.6%)	14.0 (82.4%)	38.0 (41.3%)	
Male	22.0 (73.3%)	29.0 (64.4%)	3.0 (17.6%)	54.0 (58.7%)	
Age group					0.016^1^
60–69	19.0 (63.3%)	12.0 (26.7%)	6.0 (35.3%)	37.0 (40.2%)	
70–79	9.0 (30.0%)	30.0 (66.7%)	11.0 (64.7%)	50.0 (54.3%)	
80–89	2.0 (6.7%)	3.0 (6.7%)	0.0 (0.0%)	5.0 (5.4%)	
BMI					0.678^2^
Mean (SD)	29.9 (4.7)	30.1 (4.9)	31.3 (6.6)	30.3 (5.2)	
Range	22.0–43.0	21.0–47.0	21.8–42.0	21.0–47.0	
Marital status					0.045^1^
Married	21.0 (70.0%)	39.0 (86.7%)	15.0 (88.2%)	75.0 (81.5%)	
Not married	2.0 (6.7%)	0.0 (0.0%)	0.0 (0.0%)	2.0 (2.2%)	
Divorced	1.0 (3.3%)	4.0 (8.9%)	2.0 (11.8%)	7.0 (7.6%)	
Widowed	6.0 (20.0%)	2.0 (4.4%)	0.0 (0.0%)	8.0 (8.7%)	
Education					0.043^1^
Below high school	12.0 (40.0%)	25.0 (55.6%)	10.0 (58.8%)	47.0 (51.1%)	
Diploma	5.0 (16.7%)	8.0 (17.8%)	0.0 (0.0%)	13.0 (14.1%)	
Bachelor	4.0 (13.3%)	8.0 (17.8%)	2.0 (11.8%)	14.0 (15.2%)	
High school	2.0 (6.7%)	4.0 (8.9%)	3.0 (17.6%)	9.0 (9.8%)	
Post graduate certificate	7.0 (23.3%)	0.0 (0.0%)	2.0 (11.8%)	9.0 (9.8%)	
Living with					0.025^1^
Nuclear family	27.0 (90.0%)	41.0 (91.1%)	13.0 (76.5%)	81.0 (88.0%)	
Lives alone	2.0 (6.7%)	0.0 (0.0%)	0.0 (0.0%)	2.0 (2.2%)	
Others	0.0 (0.0%)	0.0 (0.0%)	2.0 (11.8%)	2.0 (2.2%)	
Extended family	1.0 (3.3%)	4.0 (8.9%)	2.0 (11.8%)	7.0 (7.6%)	
Health status					< 0.001^1^
Excellent	6.0 (20.0%)	4.0 (8.9%)	0.0 (0.0%)	10.0 (10.9%)	
Very good	13.0 (43.3%)	12.0 (26.7%)	0.0 (0.0%)	25.0 (27.2%)	
Good	8.0 (26.7%)	17.0 (37.8%)	6.0 (35.3%)	31.0 (33.7%)	
Fair	3.0 (10.0%)	12.0 (26.7%)	10.0 (58.8%)	25.0 (27.2%)	
Poor	0.0 (0.0%)	0.0 (0.0%)	1.0 (5.9%)	1.0 (1.1%)	
Smoker					0.065^1^
Yes	1.0 (3.3%)	9.0 (20.0%)	1.0 (5.9%)	11.0 (12.0%)	
No	29.0 (96.7%)	36.0 (80.0%)	16.0 (94.1%)	81.0 (88.0%)	
Dominant hand					0.195^1^
RT	29.0 (96.7%)	42.0 (93.3%)	14.0 (82.4%)	85.0 (92.4%)	
LT	1.0 (3.3%)	3.0 (6.7%)	3.0 (17.6%)	7.0 (7.6%)	
Chronic disease					0.003^1^
No	6.0 (20.0%)	1.0 (2.2%)	0.0 (0.0%)	7.0 (7.6%)	
One	9.0 (30.0%)	12.0 (26.7%)	1.0 (5.9%)	22.0 (23.9%)	
Two	10.0 (33.3%)	11.0 (24.4%)	5.0 (29.4%)	26.0 (28.3%)	
More than 3	5.0 (16.7%)	21.0 (46.7%)	11.0 (64.7%)	37.0 (40.2%)	
Handgrip strength (kg)					0.334^1^
Within normal	12.0 (40.0%)	24.0 (53.3%)	6.0 (35.3%)	42.0 (45.7%)	
Weak	18.0 (60.0%)	21.0 (46.7%)	11.0 (64.7%)	50.0 (54.3%)	
Time Up and Go (s)					< 0.001^1^
No risk of fall	24.0 (80.0%)	21.0 (46.7%)	2.0 (11.8%)	47.0 (51.1%)	
Risk of fall	6.0 (20.0%)	24.0 (53.3%)	15.0 (88.2%)	45.0 (48.9%)	

BMI: Body Mass Index. RT: Right hand; LT: Left hand. ^1^Pearson’s Chi-squared test. ^2^Linear Model ANOVA.

There was also a strong link between living conditions and frailty (*p* = 0.025). Majority of the participants in all the groups lived in nuclear family. There was a strong link between health and frailty (*p* < 0.001). Most of the participants with frailty said their health was fair (58.8%) or good (35.3%), while most of the individuals without frailty said their health was excellent (20.0%), very good (43.3%) or good (26.7%). There was a strong link (*p* = 0.003) between the chronic disease load and the fact that 64.7% of participants with frailty had more than three chronic disorders.

Functional performance measures provided further insight. Hand grip strength showed a non-significant trend (*p* = 0.334), with the majority of participants with frailty classified as weak (64.7%). The TUG test was highly significant (*p* < 0.001), with 88.2% of participants with frailty at risk of falling, compared to only 20.0% of participants without frailty. Collectively, these findings underscore the role of gender, age, health status, chronic disease, and mobility in shaping frailty among older adults in Kuwait.

The frailty findings complement the main analysis by illustrating how frailty aligns with sarcopenia. Both conditions share common pathways of muscle loss, mobility limitation, and reduced independence, and the patterns observed here help explain how higher frailty levels correspond with increased sarcopenia risk and overall physical decline among older adults in Kuwait.

Table 5 presents the outcomes of a binomial logistic regression analysis aimed at identifying the major determinants of sarcopenia risk in older adults. The model demonstrated a satisfactory overall fit, indicated by a McFadden R^2^ of 0.333.

**Table 5:** Binomial logistic regression predicting sarcopenia risk among older adults in Kuwait (*N =* 92).

Predictor	Estimate	SE	Z	*p*	Odds ratio
Intercept	−1.461	2.397	−0.610	0.542	0.232
Gender					
Male – female	−1.670	0.690	−2.419	0.016	0.188
Age group					
60–69 – 70-79	−1.547	0.739	−2.094	0.036	0.213
80–89 – 70-79	0.512	1.312	0.3901	0.696	1.668
BMI	0.113	0.068	1.673	0.094	1.120
Education					
Diploma – below high school	−1.385	1.002	−1.382	0.167	0.250
Bachelor – below high school	−0.565	1.018	−0.555	0.579	0.569
High school – below high school	2.433	1.021	2.384	0.017	11.389
Post graduate certificate – below high school	−0.147	1.647	−0.089	0.929	0.863
Smoker					
No – yes	−1.810	0.964	−1.877	0.060	0.164
Dominant hand:					
LT – RT	0.440	1.197	0.368	0.713	1.553
Chronic diseas					
One – NO	−0.247	1.358	−0.182	0.856	0.781
Two – NO	−1.513	1.440	−1.051	0.293	0.220
More than 3 – NO	0.524	1.282	0.408	0.683	1.688

The results indicate that males were significantly less likely to experience sarcopenia compared to females (B = −1.67, SE = 0.69, z = −2.42, *p* = 0.016, OR = 0.188), suggesting that gender is a crucial predictor of sarcopenia. The age group was also significant: participants aged 60–69 years had lower odds of sarcopenia than those aged 70–79 years (B = −1.55, SE = 0.74, z = −2.09, *p* = 0.036, OR = 0.213), while the 80–89 age group did not exhibit a significant difference. The impact of education level was inconsistent. Participants who had completed high school were significantly more likely to have sarcopenia than those who had completed less than high school (B = 2.43, SE = 1.02, *p* = 0.017, OR = 11.389). The other education levels were not statistically significant. Smoking status approached significance, with non-smokers being less likely to have sarcopenia than smokers (B = −1.81, SE = 0.96, *p* = 0.060, OR = 0.164).

**Figure 1:**
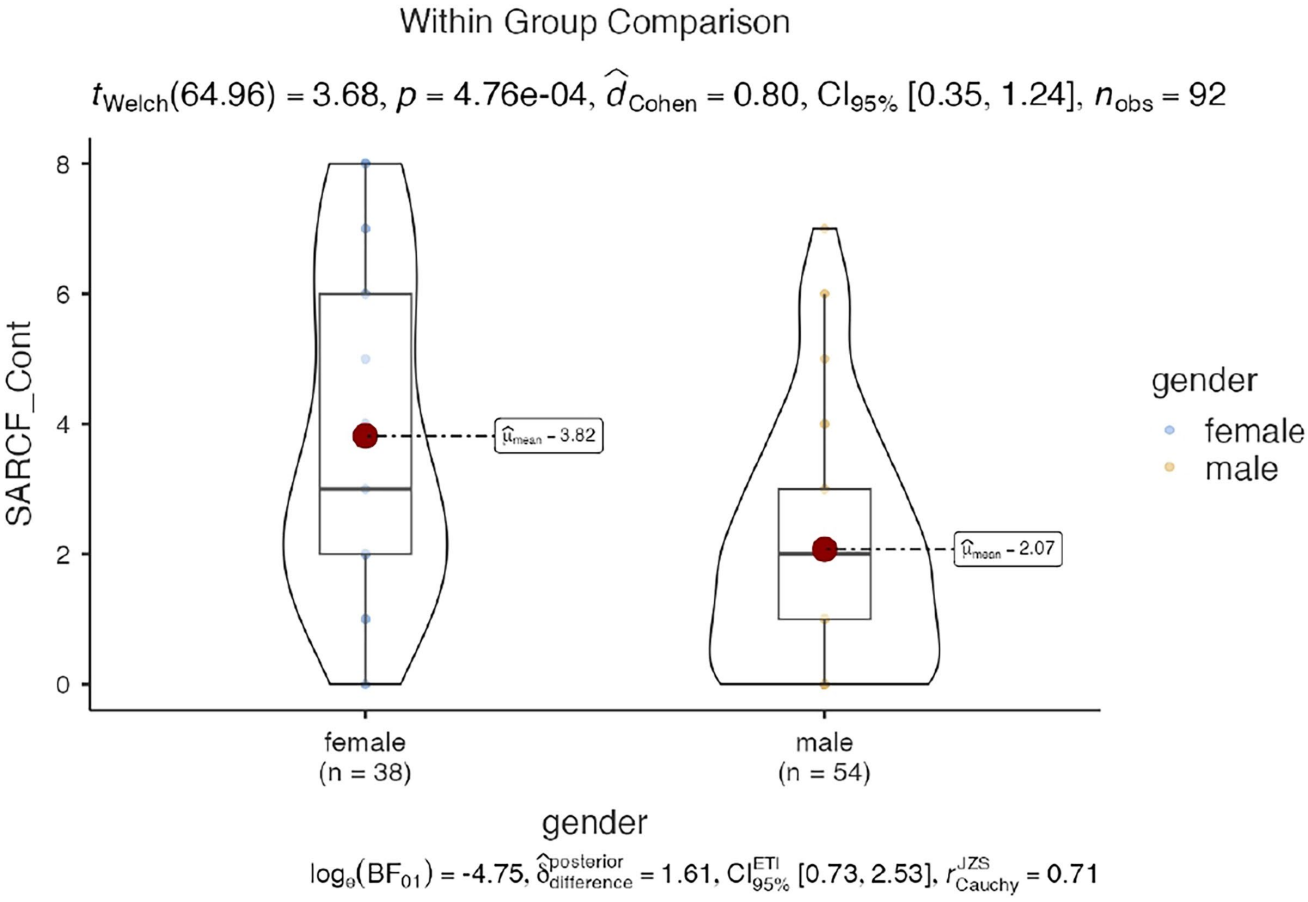
Violin plot illustrating the distribution of SARCF Category scores across gender.

**Figure 2:**
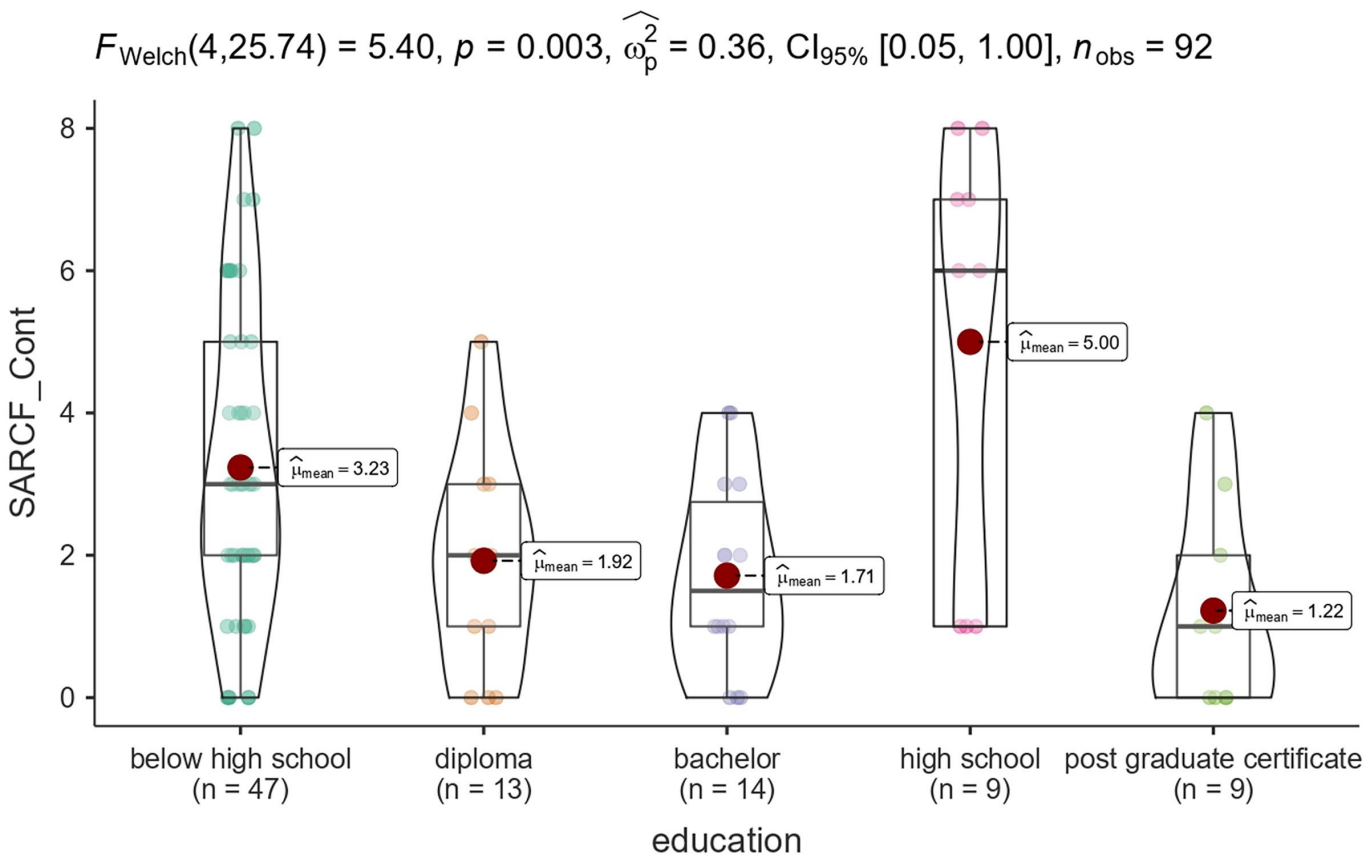
Violin plot illustrating the distribution of SARCF Category scores across education.

**Figure 3:**
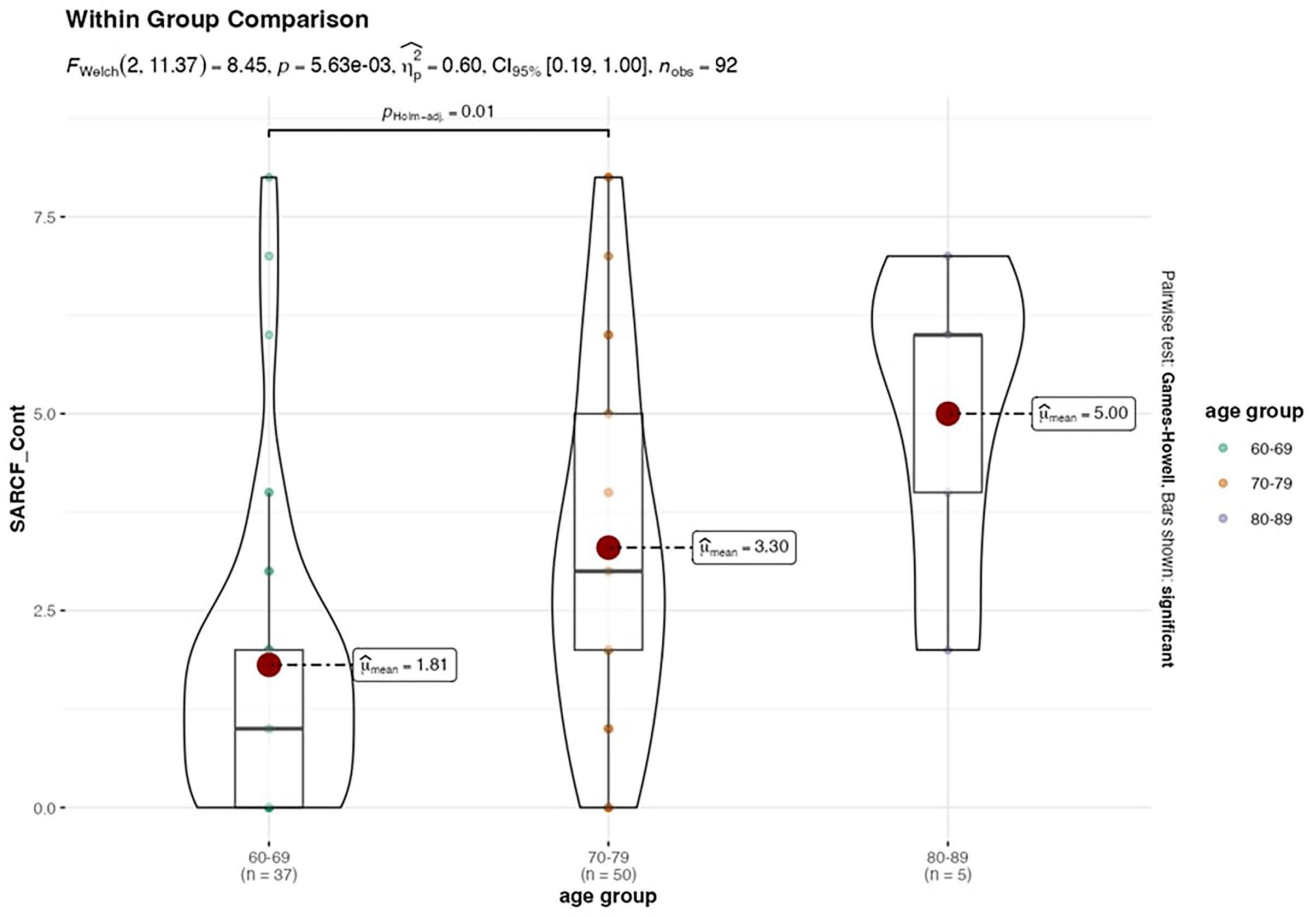
Violin plot illustrating the distribution of SARCF Category scores across age-group category.

Sarcopenia status was not significantly associated with other variables, such as dominant hand, chronic disease incidence, and recruitment source. Figures 1–3 illustrates the distribution of SARCF Category scores across gender, education and age-group category, respectively.

## 4 Discussion

This study provides the first estimate of sarcopenia risk in Kuwait, suggesting the proportion of individuals at risk may be at the higher end of global reports. Approximately one-third of community-dwelling older adults were classified as at risk of sarcopenia by SARC-F. Risk was significantly higher in females and increased with age (particularly 70–79 years). Because muscle mass was not assessed using imaging or bioimpedance techniques, a definitive diagnosis of sarcopenia could not be established. Therefore, the findings should be interpreted as reflecting risk or probable sarcopenia, rather than confirmed prevalence. Functional correlates of sarcopenia risk included slower gait speed, prolonged TUG and 5xSTS times, reduced independence in ADLs, higher FRAIL scores, and greater concern about falls. Logistic regression identified female gender and advanced age as independent predictors of sarcopenia risk. Our study also found that sarcopenia risk was associated with increased, rather than reduced, BMI. While sarcopenia risk is usually linked to lower body mass, sarcopenia obesity—where individuals with risk of sarcopenia also have overweight or obesity —has emerged (27). In our cohort, the mean BMI was 30.3 ± 5.2, placing the average participant in the obese range, reinforcing the relevance of sarcopenia obesity in this setting. The risk of obesity in Arabic-speaking countries, particularly oil-producing ones, has risen sharply over the last four decades, likely due to changes in food consumption, socioeconomic factors, and physical activity behaviours (28), contributing to risk of sarcopenia obesity in this population.

There are few reports of sarcopenia risk from Arabic countries. A study in Egypt found an 11.8% prevalence in individuals aged 60–75 based on DEXA and HGS (29), while a study from Iraq reported a 15% prevalence in adults over 50, with higher rates in males (30). We acknowledge that these studies used objective diagnostic tools, whereas our study relied on the SARC-F screening instrument. Comparisons were made only to contextualize estimates of sarcopenia risk, not to equate diagnostic precision. Differences in methodology (self-reported screening vs. imaging-based diagnosis) may account for variability in reports of sarcopenia risk, and direct comparisons should be interpreted with caution. Neither study however used SARC-F. Our finding that sarcopenia risk was greater among females aligns with a Saudi study where 71.9% of females and 59.1% of males scored ≥4 on SARC-F (31), however this such sex differences are not seen in other Asian Countries (32). The elevated female risk in Arabia may relate to cultural differences, such as traditional gender roles, physical activity behaviours and sun exposure, which affect vitamin D levels and sarcopenia risk (33). Functional associations such as impaired mobility, reduced strength, and increased fall risk, as well as links with frailty, are consistent with international consensus criteria and meta-analytic evidence demonstrating that sarcopenia is strongly associated with falls and fractures, functional decline, and mortality (1, 3, 6, 7, 10). Education status was an interesting predictor with high school graduates having greater odds of having sarcopenia when compared to that below high school (OR = 11.389) suggesting cohort-specific occupational or lifestyle pathways.

### 4.1 Practical implications

The findings of this study indicate a high risk of sarcopenia among older adults in Kuwait, underscoring the need for early screening and targeted interventions. Based on the tools used and our findings, we propose a pragmatic, physiotherapy-led screening and intervention pathway for Kuwait. Practical strategies include: (i) implementing culturally adapted Arabic versions of the SARC-F questionnaire in primary care settings for adults aged 65 years and above to identify individuals at risk; (ii) enrolling at-risk individuals in structured group exercise programs emphasizing progressive resistance training, combined with dietary counseling to ensure adequate protein intake and address vitamin D deficiency; (iii) incorporating balance training and conducting home hazard assessments for those with fear of falling or mobility impairments; and (iv) monitoring progress using standardized strength and mobility tests (e.g., handgrip strength, Timed Up and Go, Five-Times Sit-to-Stand) while collaborating with health authorities and community organizations to scale these interventions for broader public health impact.

### 4.2 Limitations and future research directions

This study has several limitations. First, a definitive diagnosis of sarcopenia could not be established because muscle mass was not quantitatively assessed using methods such as BIA, DEXA, or MRI; therefore, the findings reflect risk or probable sarcopenia rather than confirmed prevalence. Second, reliance on self-reported questionnaires may have introduced recall bias or misinterpretation, particularly given the absence of formally validated Arabic versions for some tools, including SARC-F, at the time of data collection. Third, normative reference values for the Handgrip Strength Test and the Five-Times Sit-to-Stand Test are not specific to Arabic populations, which may affect interpretation of physical performance outcomes. Fourth, the gender distribution was slightly skewed toward male participants, which may influence generalizability given known gender differences in sarcopenia risk and physical performance.

Despite these limitations, this study provides essential baseline data on sarcopenia risk and functional outcomes among older adults in Kuwait, offering valuable insights for culturally adapted screening approaches. Future research should investigate the prevalence of sarcopenia in Kuwait and aim to: (i) validate culturally adapted Arabic versions of screening tools; (ii) integrate direct measures of muscle quantity and quality (e.g., DEXA, BIA, MRI); and (iii) establish normative values for strength and mobility tests in Arabic populations. Furthermore, longitudinal studies are needed to monitor progression and evaluate intervention effectiveness, alongside in-depth investigations into sarcopenic obesity and vitamin D status.

## 5 Conclusion

The proportion of older adults at risk of sarcopenia in Kuwait appears substantial. While our findings reflect risk rather than confirmed prevalence, they highlight the clinical utility of simple screening and functional tests to identify vulnerable individuals. Integrating objective muscle mass assessments and culturally adapted tools into future studies will improve diagnostic accuracy and guide scalable screening and rehabilitation services for Kuwait’s aging population.

## Data Availability

The raw data supporting the conclusions of this article will be made available by the authors, without undue reservation.
